# The anticancer mechanisms of *Toxoplasma gondii* rhoptry protein 16 on lung adenocarcinoma cells

**DOI:** 10.1080/15384047.2024.2392902

**Published:** 2024-08-22

**Authors:** Guangqi Li, Qinhui Li, Yongqing Tong, Jin Zeng, Tiantian Dang, Ningai Yang, Yuning Zhou, Lei Ma, Qirui Ge, Zhijun Zhao

**Affiliations:** a Medical Laboratory Center, General Hospital of Ningxia Medical University, Yinchuan, China; b Ningxia Key Laboratory of Clinical Pathogenic Microorganisms, General Hospital of Ningxia Medical University, Yinchuan, China; c Ningxia Clinical Research Center of Medical Laboratory, General Hospital of Ningxia Medical University, Yinchuan, China; d College of Life Sciences, Ningxia University, Yinchuan, China; e Key Laboratory of Ministry of Education for Conservation and Utilization of Special Biological Resources in the Western, Ningxia University, Yinchuan, China; f Department of Clinical laboratory, Renmin Hospital of Wuhan University, Wuhan, China; g Institute of Medical Sciences, General Hospital of Ningxia Medical University, Yinchuan, China; h The First Clinical Medical College, Ningxia Medical University, Yinchuan, China

**Keywords:** Toxoplasma gondii, ROP16, lung cancer, A549, cell phenotype, STAT3

## Abstract

Lung adenocarcinoma is the most prevalent subtype of lung cancer, which is the leading cause of cancer-related mortality worldwide. *Toxoplasma gondii* (*T.gondii*) Rhoptry protein 16 (ROP16) has been shown to quickly enter the nucleus, and through activate host cell signaling pathways by phosphorylation STAT3 and may affect the survival of tumor cells. This study constructed recombinant lentiviral expression vector of *T. gondii* ROP16 I/II/III and stably transfected them into A549 cells, and the effects of ROP16 on cell proliferation, cell cycle, apoptosis, invasion, and migration of A549 cells were explored by utilizing CCK-8, flow cytometry, qPCR, Western blotting, TUNEL, Transwell assay, and cell scratch assay, and these effects were confirmed in the primary human lung adenocarcinoma cells from postoperative cancer tissues of patients. The type I and III ROP16 activate STAT3 and inhibited A549 cell proliferation, regulated the expression of p21, CDK6, CyclinD1, and induced cell cycle arrest at the G1 phase. ROP16 also regulated the Bax, Bcl-2, p53, cleaved-Caspase3, and Caspase9, inducing cell apoptosis, and reduced the invasion and migration of A549 cells, while type II ROP16 protein had no such effect. Furthermore, in the regulation of ROP16 on primary lung adenocarcinoma cells, type I and III ROP16 showed the same anticancer potential. These findings confirmed the anti-lung adenocarcinoma effect of type I and III ROP16, offering fresh perspectives on the possible application of ROP16 as a target with adjuvant therapy for lung adenocarcinoma and propelling the field of precision therapy research toward parasite treatment of tumors.

## Introduction

Lung cancer is a major threat to human health worldwide, being the leading cause of cancer-related death and the second most common cancer type. In 2020 alone, there will be approximately 220,000 lung cancer cases and 179,000 deaths globally.^1^ There are two main pathological types of lung cancer: small-cell lung cancer (SCLC) and non-small cell lung cancer (NSCLC), NSCLC accounts for 85% of all lung cancers. Lung adenocarcinoma (LUAD) is the most prevalent subtype of NSCLC, accounting for about 40% of all lung cancer cases.^2^ LUAD has a poor prognosis due to the presence of micro-metastatic lung lesions, which often lead to early metastasis.^3^ Current standard treatment modalities of surgery, radiation, and chemotherapy all have certain limitations in the treatment of patients with advanced metastatic LUAD.^3^ In recent years, targeted therapy and immunotherapy have become hot spots in the treatment of malignant tumors. Targeted therapy is a high-precision treatment option that has become the standard for patients with sensitive gene mutations.^4^ However, further research is still needed for targeted therapy due to the resistance genes of tumors, tumor heterogeneity, and specificity of targets. Immunotherapy has become another therapeutic method that can improve the sensitivity of the body’s immune system to tumor cells and kill them specifically.^5^ Promising prospects have been observed for immune checkpoint inhibitors such as programmed death receptor 1 (PD-1)/programmed death ligand (PD-L1) inhibitors.^6–8^ However, further research is needed to address issues such as hyperimmune response, pseudoprogression, and the complexity and diversity of tumor microenvironments. Therefore, the search for new antitumor therapeutic and other adjuvant therapies means has become a research focus in the treatment of LUAD.

According to most studies, parasites have been detected in the bodies of many cancer patients, as research on the relationship between parasites and tumors deepens, an increasing number of correlations have been found between the two, and certain parasites have been shown to have anti-tumor effects.^9–11^ In recent times, a number of live-attenuated protozoan parasites, such as *Leishmania infantum* and *L. tropica*,^12^ *Neospora caninum*,^13^ and *T.gondii*,^14^ have been shown to be a potential biological agents for the treatment of tumors through intratumoral injection. Studies using mouse cancer models have demonstrated that parasitic infections can activate the immune system suppressed by cancer cells, counteract the tumor’s immunosuppressive microenvironment, inhibit tumor angiogenesis, growth, metastasis, and prolong the survival time of tumor-bearing mice.^15^ With studies reporting that parasitic secretion antigens and proteins of *T.gondii* can induce apoptosis and inhibit proliferation in various cancer cell lines, including ovarian,^16^ hepatoma,^17^ neuroblastoma,^18^ and human neuroblastoma.^19^ However, no studies have yet investigated the effect and mechanism of *T. gondii* ROP16 on lung adenocarcinoma.

*T.gondii* is a zoonotic parasite with a high infection rate and low morbidity, capable of infecting almost all warm-blooded vertebrates. It is estimated that up to one-third of the world’s population is chronically infected with *T.gondii*, which can cause chorioretinitis, encephalitis, pulmonary toxoplasmosis, and other severe diseases, particularly in immunodeficient patients who receive cancer chemotherapy, organ transplantation, or those infected with human immunodeficiency virus (HIV). *T.gondii* invades the human body and quickly spreads to multiple organs, and its tachyzoite stage may form cysts to induce chronic or latent infection in the brain, heart, skeletal muscle, and other organs. *T.gondii* has fifteen genetic haplotypes, of which types I, II, and III are the typical genotypes, and the virulence of *T. gondii* is strain-specific and closely related to rhoptry protein, dense granular protein, and microneme protein.^20^ Rhoptry is an essential organelle for the normal growth and metabolism of *T.gondii* and plays a critical role in the pathogenesis of the parasite, invasion of host cells, and interaction with host cells.^21^ Rhoptry proteins are critical for T. gondii survival in host cells and are involved in invasion, the creation of the parasite vacuole replication niche, host cell manipulation, and innate immunity.^22^ Rhoptry protein 16 (ROP16) is an essential virulence factor that controls the transmission of signals from host cells, localizes directly to the host nucleus, and is essential for immune evasion by host cells.^23^ ROP16 has been demonstrated to upregulate the synthesis of proinflammatory IL-12 and increase the production of interleukin-4 (IL-4) in type I and III genotypes.^24^ What’s more, the researchers have found that the type I and III genotypes ROP16 protein affects different signaling pathways by activating STAT3/6 after the invasion of host cells,^25^ which leads to more roles of these two ROP16 proteins in host cells.

In this study, we utilized gene engineering techniques to construct recombinant *T. gondii* ROP16 I/II/III lentiviral vectors, which were stably transfected into A549 lung adenocarcinoma cells. Our investigation focused on the effects of ROP16I/II/III on cell apoptosis, cell cycle, proliferation, invasion, and migration of A549 cells. Furthermore, we explored the influence of ROP16 I/II/III on the expressions of cell cycle-related and apoptotic-related proteins, as well as STAT3 phosphorylation, to identify the signaling pathways that are regulated by ROP16 to inhibit cancer proliferation. To verify the function of different genotypes ROP16 in real tissues, we constructed the ROP16I/II/III overexpression system in primary lung adenocarcinoma cells and performed functional validation. The findings of this study will provide fundamental evidence for adjuvant therapy with parasite-secreted proteins in lung adenocarcinoma and may indicate promising and effective targeted therapeutic molecules for personalized medicine in lung cancer.

## Results

### Transfection of A549 cells with lentiviral vector overexpressing ROP16 I/II/III

**Figure 1.**
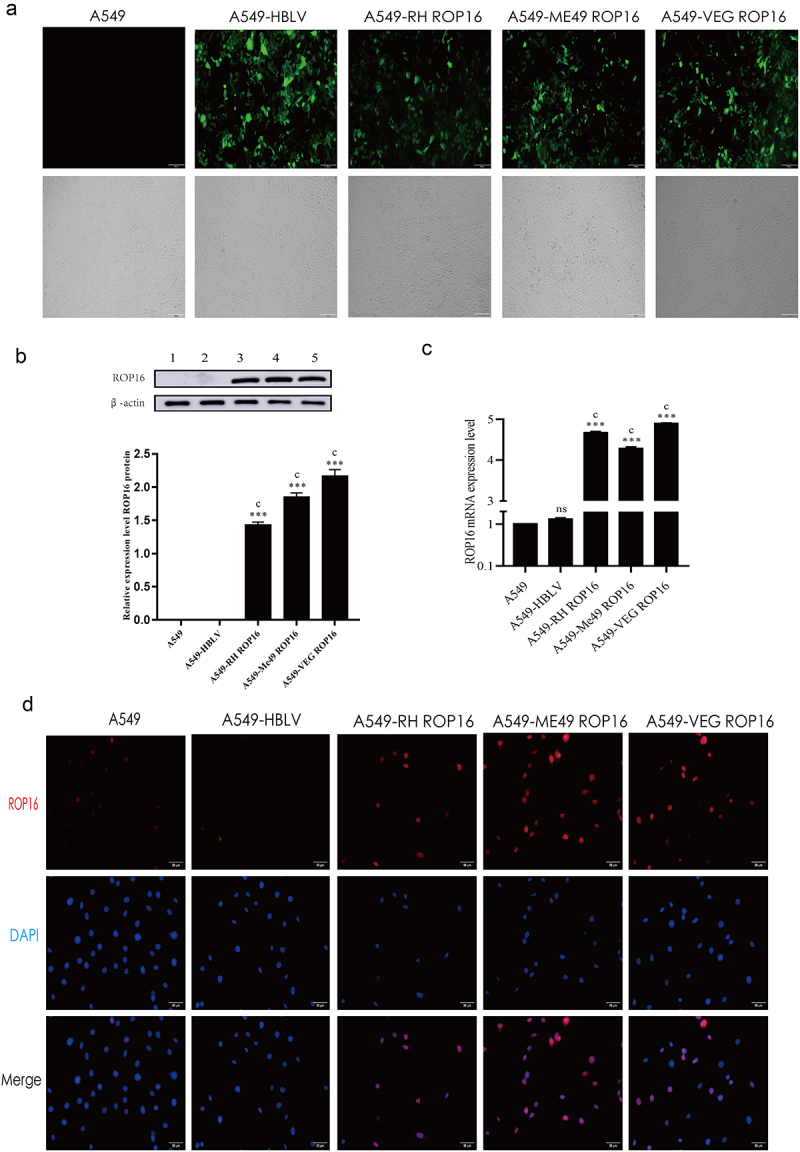
Transfection of A549 cells with lentiviral vector overexpressing ROP16 I/II/III. (a) Fluorescence image of A549 cells transfected with lentivirus overexpressing *rop*16I/II/III (b) The expression of ROP16 protein in A549 was detected by Western Blotting and the grayscale statistical analysis of this detection results; (c) The mRNA expression of ROP16 in A549 was detected by RT-PCR. (d) Immunofluorescence detection of ROP16 expression in A549 cells(400×). The results were given as mean ± SD and were analyzed by one-way ANOVA, compared with blank group, *P <.05,**P <.01,***P <.001; compared with overexpression control , a P <.05, b P <.01, c P <.001.

A recombinant vector overexpressing *T. gondii* ROP16 RH (type I), Me49 (type II), and VEG (type III) was constructed using gene engineering techniques and identified through gene sequencing. Lentiviral vectors containing plasmids pSPAX2, pMD2G, and the shuttle plasmid were constructed and proliferated in 293T cells. Successful construction of the recombinant vector was confirmed by consistency of the target gene and testing sequence. The constructed vector was then transfected into 293T cells for amplification. A549 cells were transfected with the RH-ROP16, Me49-ROP16, VEG-ROP16 overexpression lentivirus at an appropriate MOI, and transfection efficiency was determined by measuring the expression of green fluorescent protein (GFP). The results indicated that the recombinant vector had been successfully transfected into A549 cells (Figure 1a).

Stably transfected A549 cells overexpressing ROP16 were screened using puromycin over a period of two weeks. Expression of three genotypes ROP16 protein (Figure 1b) and mRNA (Figure 1c) were significantly higher in the ROP16 overexpression groups compared to the blank control (A549) and empty vector control (A549-HBLV), with some differences among the three genotypes of A549 cells (i.e., ROP16 type I, type II, and type III). Immunofluorescence staining revealed that the green fluorescence was distributed in the cytoplasm as well as in the nucleus 72 h after transfection (Figure 1d).

### Influences of T. gondii ROP16 on the phenotype of A549 cells

#### Effects of ROP16 on cell proliferation of A549 cells

**Figure 2.**
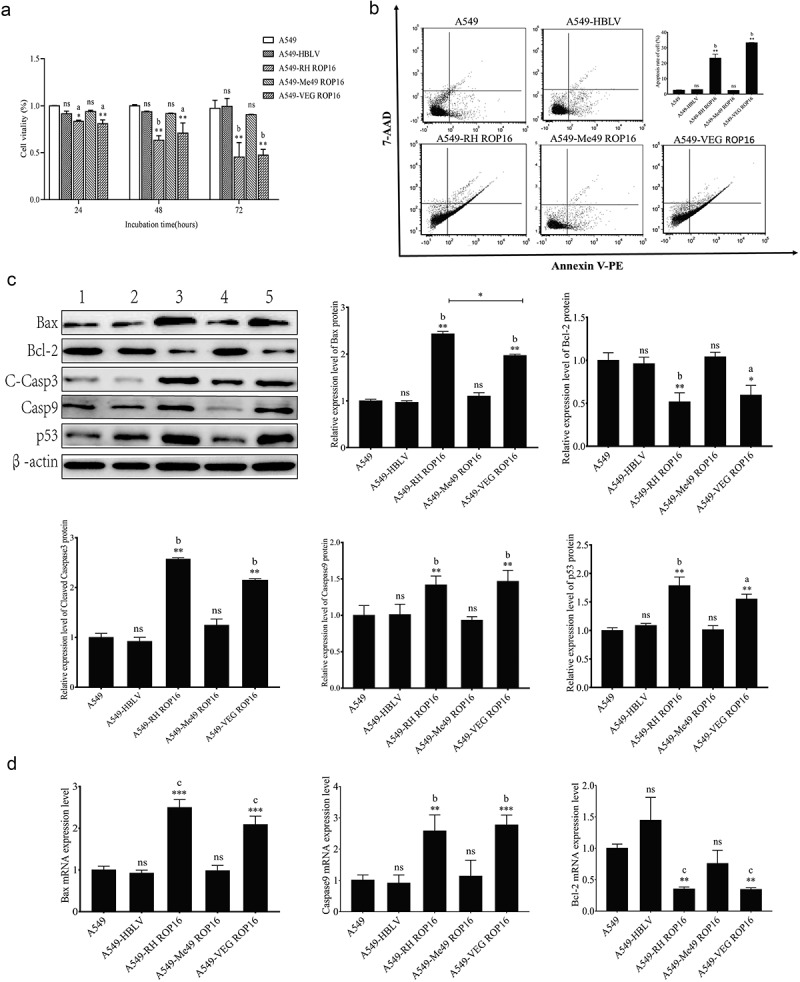
Effects of ROP16 on cell proliferation and apoptosis of A549 Cells (a) statistical chart of proliferation of A549 cells after ROP16 overexpression detected by CCK-8(±s). (b) Flow cytometry analysis of the effect of ROP16 overexpression on apoptosis of A549 cells and statistical analysis of apoptosis. (c) The expression of Bax,Bcl-2,C-Casp3,Casp9,p53 protein in A549 was detected by Western Blotting and the grayscale statistical analysis of this detection results. Compared with blank group, *P <.05,**P <.01,***P <.001; compared with overexpression control , a P <.05, b P <.01, c P <.001.

The impact of *T. gondii* ROP16 on A549 cell proliferation was analyzed using the CCK-8 (Figure 2a). After 24 hours of culture, the cell proliferation of A549-RH ROP16 cells was inhibited (*p* < .05), and cellular activity was reduced by 20%, and after transfection for 48 hours and 72 hours (*p* < .01), the cellular activity was reduced by 37% and 55%, respectively. However, the proliferation of A549-Me49 ROP16 cells was not significantly influenced after culture for 24 hours or longer (*p* > .05). The proliferation of A549-VEG ROP16 cells was significantly inhibited after culture for 24 hours, 48 hours, and 72 hours (*p* < .01). These findings suggest that ROP16I and ROP16III significantly inhibit A549 cell proliferation, while ROP16II has no significant effect on A549 cell proliferation.

#### Effects of ROP16 on apoptosis of A549 cells

The effect of *T. gondii* ROP16 on A549 cell apoptosis was analyzed by cytometry after 48 hours of culture (Figure 2b). The results showed that cell apoptosis increased from 2.62% to 23.31% in A549-RH ROP16 and 33.31% in A549-VEG ROP16, respectively, compared to the non-transfected group (*p* < .01). No significant difference was found for apoptosis of A549-Me49 ROP16 cells (*p* > .05). Western blotting analysis showed that, compared with the non-transfected group and the empty vector control group, the expression of the pro-apoptotic protein Bax was significantly up-regulated and the expression of the anti-apoptotic protein Bcl-2 was significantly down-regulated in the RH and VEG ROP16 (F = 63.97, *p* < .01). There was no significant difference between the Me49 ROP16 overexpression group and the non-transfection group (*p* > .05), indicating that RH and VEG ROP16 could promote apoptosis of A549 cells, but Me49 ROP16 had no significant effect. Furthermore, the results showed that p53, Caspase9, and CleavedCaspase3 proteins were significantly up-regulated in the RH and VEG ROP16 overexpression group compared with the non-transfected and the empty vector controls (F = 63.97, *p* < .01) (Figure 2c). Quantitative Real-time PCR was used to detect the mRNA of apoptosis-related proteins in each group, and the results were consistent with the results of previous experiments (Figure 2d). In conclusion, typeI and III ROP16 promote A549 cell apoptosis, while type II ROP16 protein has no significant effect on A549 cellular apoptosis.

**Figure 3.**
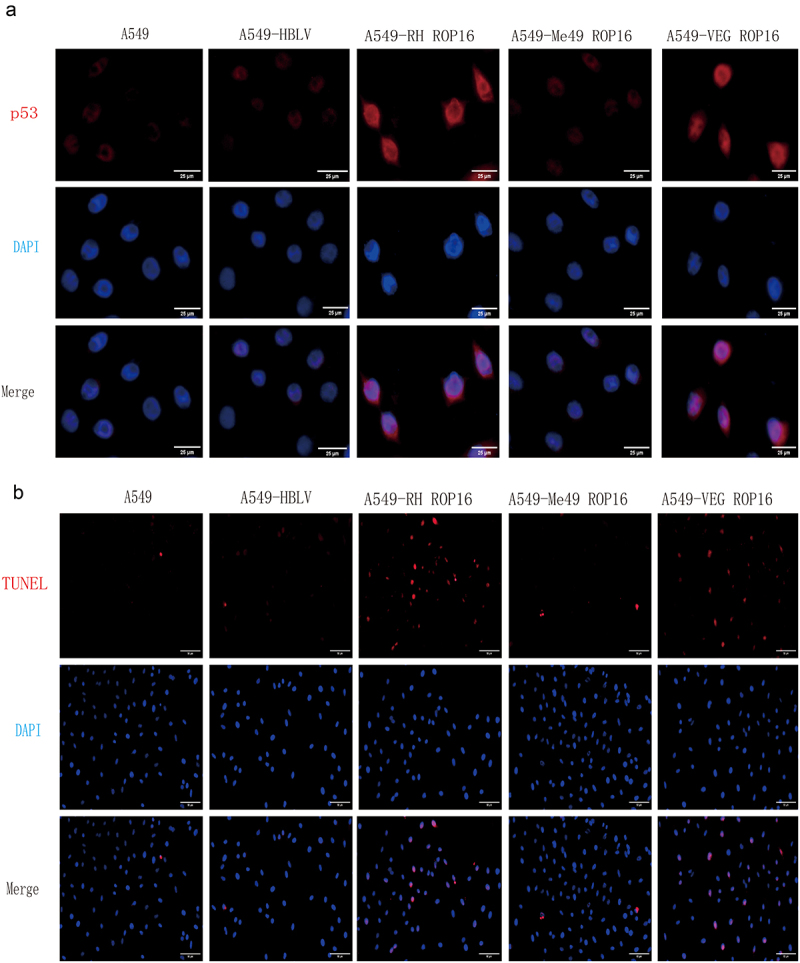
Immunofluorescence assay to detect the effect of rop16 on apoptosis in A549 cells. (a) Immunofluorescence detection of the effect of overexpression of ROP16 on the expression of p53 protein in A549 cells. (b) TUNEL assay detects the effect of overexpression of ROP16 on apoptosis of A549 cells(100×).

Subsequently, we evaluated the expression level of p53 by immunofluorescence and found that both type I and type III ROP16 induced the expression of apoptosis-related proteins in A549 cells, leading to cell apoptosis (Figure 3a). To investigate the relationship between ROP16 and A549 cell apoptosis further, we used TUNEL staining to analyze the effect of ROP16 on A549 cell apoptosis. Our results showed (Figure 3b) that after 48 hours of culture, the rate of TUNEL-positive cells was significantly increased in type I and type III ROP16 overexpression groups (*p* < .01). However, compared with the non-transfected group, the rate of TUNEL-positive cells in the control group was not statistically significant (*p* > .05). The TUNEL-positive cell rate of type II ROP16 overexpression group did not exhibit a significant change (*p* > .05). These results are consistent with the above findings.

#### Effects of ROP16 on cell cycle of A549 cells

**Figure 4.**
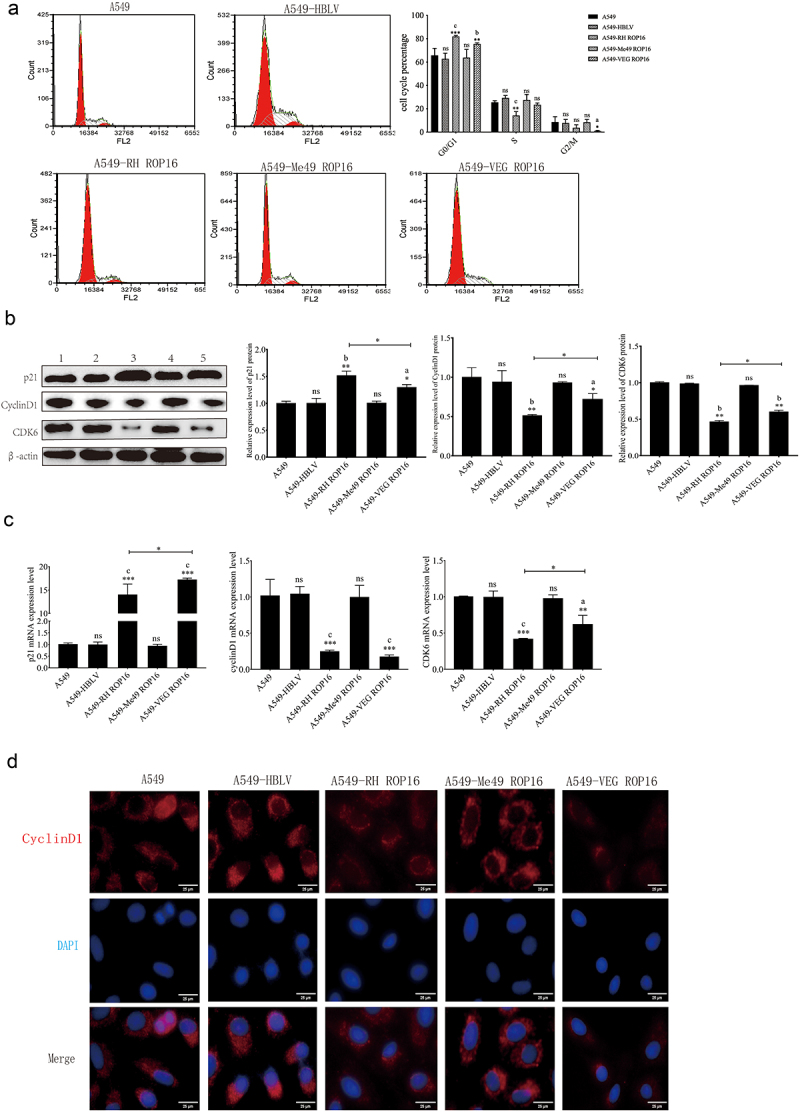
Effects of ROP16 on cell cycle of A549 cells. (a) Flow cytometry detection of changes in A549 cell cycle after ROP16 overexpression. (b) The expression of p21,CycilnD1,CDK6 protein in A549 was detected by Western Blotting and the grayscale statistical analysis of this detection results. (c) the effect of ROP16 protein on p21, CDK6 and CyclinD1 mRNA expression in A549 cells was detected by q-pcr. (d) The effect of ROP16 overexpression on CyclinD1 protein expression in A549 cells was detected by immunofluorescence.(400×). Compared with blank group, *P <.05,**P <.01,***P <.001; compared with overexpression control , a P <.05, b P <.01, c P <.001.

Furthermore, we analyzed the effect of ROP16 on cell cycle in A549 cells using Flow cytometry. We observed changes in the cell cycle of overexpression RH ROP16 cells, with an increasing proportion of G0/G1 phase cells (from 63.8% to 82.0%) (*p* < .05) and a decreasing proportion of S phase cells (from 25.5% to 14.3%) (*p* > .05). The proportion of G2/M phase cells also decreased, but not significantly (*p* > .05). Overexpression VEG ROP16 cells showed a significant increase in the proportion of G0/G1 phase cells to 75.6% (*p* < .05), which was similar to the trend observed in RH ROP16 cells group. The proportion of S and G2/M phase cells decreased in RH ROP16 cells, with significant changes in G2/M phase cells (*p* < .05). However, overexpression Me49 ROP16 cells group non-transfected group. Our findings from flow cytometric analysis suggest that type I and type III ROP16, but not type II ROP16, regulate cell cycle progression in A549 cells (Figure 4a).

Moreover, we used Western blotting and Immunofluorescence to detect the effect of ROP16 on A549 cell cycle-related protein expression to verify and explore the relationship between ROP16 and A549 cell cycle arrest. Western blotting analysis of G1 phase-related protein expression in each group A549 cells showed that compared with the control group, the expression of the cell cycle suppressor protein p21 was up-regulated in RH and VEG ROP16 overexpression groups (*p* < .05). The expressions of CDK6 and CyclinD1 were significantly down-regulated (*p* < .05), but there was no significant difference between the overexpression control group and the Me49 ROP16 overexpression group (*p* > .05) (Figure 4b).

In addition, the expression of G1 phase-related mRNA was detected by qPCR. The results showed (Figure 4c) that compared with non-transfected group, the expression of p21 mRNA was significantly up-regulated in RH and VEG ROP16 overexpression groups (*p* < .05), while the expressions of CDK6 mRNA and CyclinD1 mRNA were significantly down-regulated (*p* < .05). These results indicated that RH and VEG ROP16 regulated the expression of A549 cell cycle G1 phase related mRNA, while Me49 ROP16 had no significant effect on the expression of cell cycle-related mRNA, which was consistent with the above Flow detection and Western blotting results.

Nextly,we detected the expression level of CyclinD1 by Immunofluorescence, which was consistent with the results of previous experiments (Figure 4d). These results indicated that type I and type III ROP16 regulated the expression of G1 phase protein of A549 cell cycle, while type II ROP16 had no significant effect on the expression of A549 cell cycle protein.

#### Effects of ROP16 on invasion and migration of A549 cells

**Figure 5.**
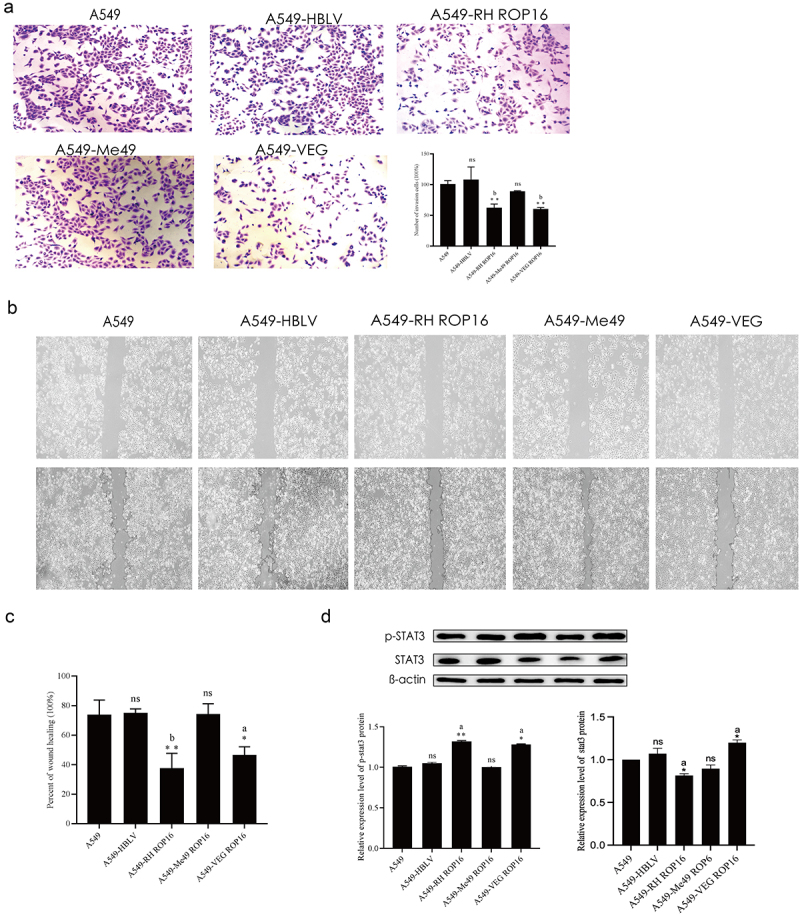
Effects of ROP16 on invasion, migration, and STAT3 phosphorylation of A549 cells. (a) Transwell assay detects changes in the invasive ability of A549 cells after ROP16 overexpression (100×). Graph of statistical analysis of cell invasion. (b) Cell scratch assay to detect the effect of ROP16 overexpression on the migration ability of A549 cells (100×). (c) Quantification of the percentage of healing. (d) Western blotting detection of p-STAT3 protein expression in A549 after ROP16 overexpression and statistical analysis of assay results. Compared with blank group, *P <.05,**P <.01,***P <.001; compared with overexpression control , a P <.05, b P <.01, c P <.001.

The effects of *T. gondii* ROP16 on invasion of A549 cells were investigated through Transwell cell viability assay. A549 cells transfected with three genotypes ROP16 and control group cells at logarithmic growth phase were harvested for Transwell assay (Figure 5a). Compared to that of control group, overexpression RH and VEG ROP16 cells showed significantly reduced invasion ability (*p* < .01), but overexpression control group and the Me49 ROP16 overexpression group cells did not show significant difference in invasion ability (*p* > .05). These data indicated that only type I and III ROP16 have the effect of invasion inhibition of lung adenocarcinoma A549 cells.

Wound-healing assay investigated the effects of ROP16 on migration of A549 cells. Wound was produced by sterile tips on the surface of plate with cultured cells, and cell migration distance was detected after 48 h, as shown in Figure 5b. The proportion of wound-healing was then quantified, as shown in Figure 5c. These data showed that control group and the type II ROP16 overexpression group cells did not show significant alteration in cell migration distance when compared to that of blank control (*p* > .05). Type I (*p* < .01) and type III (*p* < .05) ROP16 have the effect of migration inhibition of A549 cells, and typeI ROP16 may have a higher ability of this migration inhibition.

#### Regulatory mechanisms of ROP16 on the A549 cells

It has been found that there is a serine/threonine kinase region in the ROP16 protein sequence that can exert kinase activity, participate in regulating the activation process of STAT3 and STAT6 in host cells, and induce their phosphorylation, meanwhile the phosphorylation of STAT3 (p-STAT3) and STAT6 (p-STAT6) activates the downstream modulators.^26^ In our experiment, Western blotting results showed that p-STAT3 levels in type I ROP16 and type III ROP16 cells had significantly increased (*p* < .05), while p-STAT3 levels in type II ROP16 cells had not significantly changed (*p* > .05) compared to the control. The data was shown in Figure 5d.

### Verification of overexpression ROP16 I/II/III on lung adenocarcinoma primary cells

#### Transfection with overexpression ROP16 lentivirus for primary lung adenocarcinoma cells

**Figure 6.**
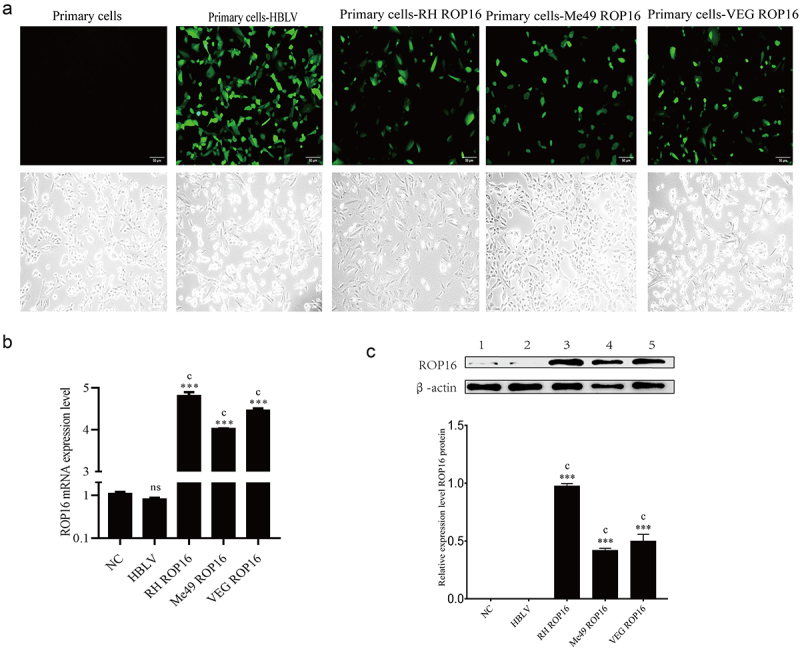
Transfection with overexpression ROP16 lentivirus for primary lung adenocarcinoma cells. (a) The 48 h fluorescence images of primary lung adenocarcinoma cells transfected with lentivirus overexpressing rop16(100×). (b) The expression of ROP16 protein in A549 was detected by Western blotting and its grayscale statistical analysis; (c) The mRNA expression of ROP16 in A549 was detected by RT-PCR. Compared with blank group, *P <.05,**P <.01,***P <.001; compared with overexpression control , a P <.05, b P <.01, c P <.001.

We collected and separated lung adenocarcinoma primary cells, which were cultured routinely for 48 hours and then transfected transiently with ROP16 I/II/III lentiviral vectors.GFP was quantified to evaluate the transfection level of recombinant vectors in lung adenocarcinoma primary cells. It was indicated that a large amount of GFP could be seen in ROP16-transfected cells, including control group, type I, type II, and type III ROP16 overexpression lung adenocarcinoma primary cells, as shown in Figure 6a. Relative expression of *rop*16 mRNA was determined by qPCR (Figure 6b), and expression of ROP16 protein was determined by Western blotting using the primary lung adenocarcinoma cells after 48 hours of cell culture (Figure 6c). *Rop*16 mRNA expression was increased, and ROP16 protein expression was also upregulated in the three genotypes of ROP16 compared to the control. These data indicated that recombinant *T. gondii* ROP16 was successfully transfected into lung adenocarcinoma primary cells.

#### Regulation of ROP16 on expression of apoptotic-related proteins in primary lung adenocarcinoma cells

**Figure 7.**
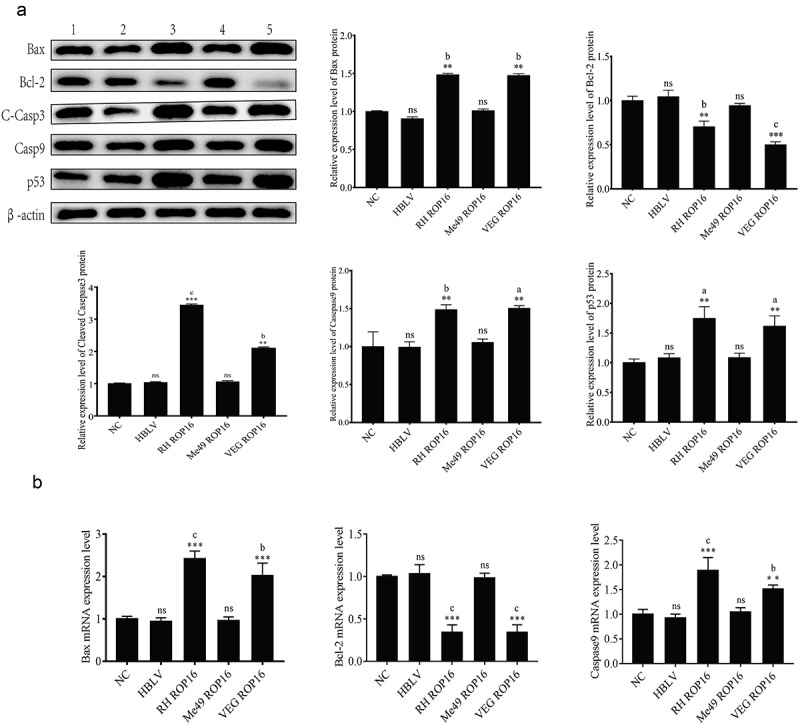
Effects of ROP16 on cell apoptosis of lung adenocarcinoma primary cells. (a) The expression of Bax,Bcl-2,C-Casp3,Casp9,p53 protein in primary lung adenocarcinoma cells was detected by Western Blotting and the grayscale statistical analysis of this detection results. (b) The effect of ROP16 protein on Bax and bcl-2, Caspase9 mRNA expression in primary lung adenocarcinoma cells was detected by q-pcr and statistical analysis of q-pcr assay results. Compared with blank group, *P <.05,**P <.01,***P <.001; compared with overexpression control , a P <.05, b P <.01, c P <.001.

The expressions of apoptotic-related proteins, such as Bax, Bcl-2, p53, Caspase9, and Cleaved Caspase3, were analyzed through Western blotting. The results showed that the control group and type II ROP16 did not exhibit significant alterations compared to the blank control (*p* > .05). However, type I and type III ROP16 primary lung adenocarcinoma cells showed a significant upregulation of pro-apoptotic protein Bax and downregulation of anti-apoptotic protein Bcl-2 compared to that of the control (*p* < .01). Moreover, p53, Caspase9, and Cleaved Caspase3 were significantly upregulated in type I and type III ROP16 primary lung adenocarcinoma cells compared to that of the control (*p* < .05). These data are presented in Figure 7a. To confirm these results, the expression of apoptosis-related mRNA was analyzed through qPCR (Figure 7b). The results showed that the expression of Bax mRNA and Caspase9 mRNA was significantly upregulated (*p* < .01), while that of Bcl-2 mRNA was significantly downregulated (*p* < .01) in the type I and type III ROP16 overexpression groups.

#### Influence of ROP16 on cell cycle-related proteins of primary lung adenocarcinoma cells

**Figure 8.**
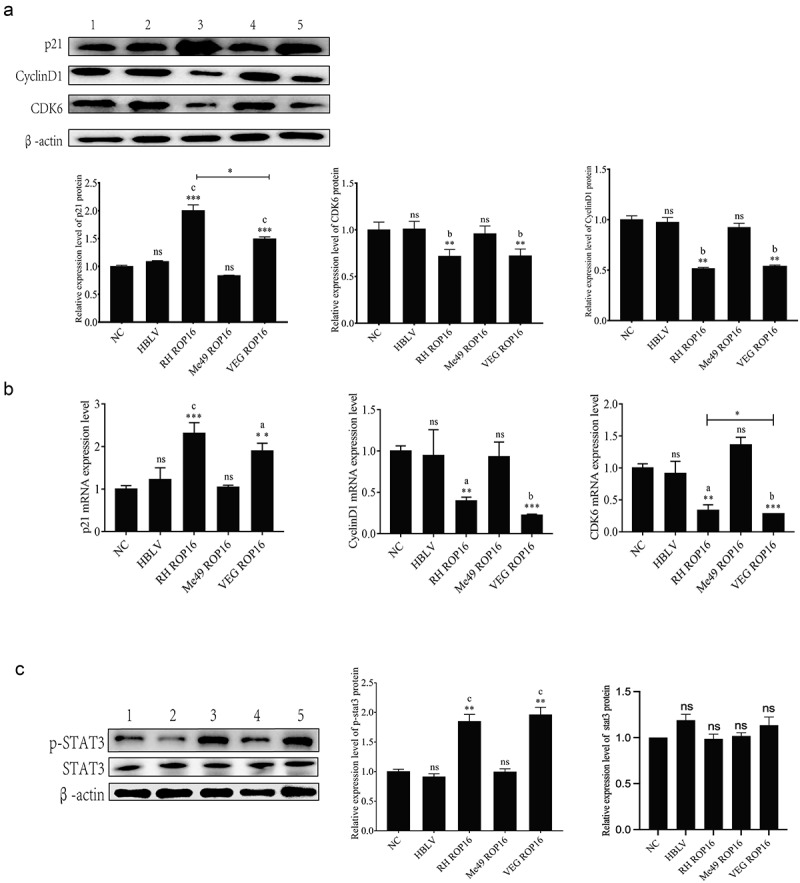
Effects of ROP16 on cell cycle of lung adenocarcinoma primary cells. (a) The expression of p21, CyclinD1, and CDK6 protein in primary lung adenocarcinoma cells was detected by Western Blotting and the grayscale statistical analysis of this detection results. (b) The effect of ROP16 protein on p21,CyclinD1 and CDK6 mRNA expression in primary lung adenocarcinoma cells was detected by q-pcr and statistical analysis of q-pcr assay results. (c) The expression of p-STAT3 protein in primary lung adenocarcinoma cells after ROP16 overexpression was detected by Western Blotting and statistical analysis of assay results. Compared with blank group, *P <.05,**P <.01,***P <.001; compared with overexpression control , a P <.05,b P <.01,c P <.001.

In type I and type III ROP16 groups, the expression of p21 was significantly increased, while CDK6 and CyclinD1 were significantly decreased compared to that of the control (*p* < .01). Moreover, there was a significant difference in p21 protein expression between type I and type III ROP16 cells (Figure 8a). Further validating the expression of G1 phase-associated mRNAs by qPCR. The results were consistent with Western blotting (*p* > .05) (Figure 8b).

#### Activation of ROP16 on STAT3 of primary lung adenocarcinoma cells

In three genotypes of ROP16 overexpressing primary lung adenocarcinoma cells, we observed similar results to A549 for the phosphorylation of STAT3 by ROP16. Type I and type III ROP16 overexpression primary lung adenocarcinoma cells expressed increased p-STAT3 compared to that of the control (*p* < .01), but this was not observed in type II ROP16 overexpression group (*p* > .05) (Figure 8c).

## Discussion

Lung cancer is the most frequent cause of cancer-related deaths worldwide, In 2020 only, there will be 2 million new cases of lung cancer and 1.8 million deaths worldwide. Globally, lung cancer is the leading cause of cancer deaths in men and the second leading cause of cancer deaths in women, after breast cancer. Depending on the stage, differences in smoking patterns, and trends in economic development, 5-year survival rates range from 4% to 17%.^27^ It is a complex disease, and its progression is influenced by a variety of factors. In recent years, with the continuous discovery of NSCLC pathogenic genes and gene polymorphisms related to pathology, physiology, and metabolism, as well as the rapid development of histopathology and sequencing technologies, the foundation for precision treatment of NSCLC has been established. However, the current therapies for advanced NSCLC patients still have many limitations such as chemotherapy resistance, targeted drug resistance, and near- and long-term complications of radiotherapy. Precision therapy for NSCLC patients still requires accurate genetic testing and is not effective in prolonging overall survival (OS) and Progression-Free Survival (PFS). Currently, only 20% to 50% of patients can benefit from precision treatment, even for patients who express positive immune checkpoints, 40% to 50% cannot benefit from immunotherapy.^28^ Studies have found that gender, age, and smoking status are the main sociodemographic factors affecting the effectiveness of precision treatment for NSCLC. In addition, tumor TNM staging, tgr, pre-treatment physical condition, pathological type, underlying diseases, and the use of other therapeutic drugs also have certain impacts on the effectiveness of precision treatment.^5,29^ Moreover, the driver gene targets in targeted therapy, immune checkpoints in immunotherapy, intratumoural immune cell infiltration, intratumoural immune cell infiltration, peripheral blood and serological indicators, tumor mutation burden, and lung immune prognostic index all have important impacts on the treatment and prognosis of NSCLC.^5^ Therefore, analyzing the different treatment effects produced by different influencing factors and conducting better and more precise diagnoses, and continuous research and development of drugs and treatments for NSCLC can help improve the efficacy and prognosis of NSCLC patients.

*T. gondii* is a specialized intracellular parasite belonging to the apicomplexan. It has a complex life-cycle, with cats and felines as its final hosts, and almost all warm-blooded animals can be its intermediate hosts. During parasitization by *T. gondii*, the host develops a series of stress responses, and under these unfavorable conditions *T. gondii* switches from the active tachyzoite state to the dormant bradyzoite state, which is difficult to be recognized by the immune system and exogenous drugs in the host. However, when the host develops other diseases or is immunocompromised, the surrounding environment becomes suitable for its reproduction, and *T. gondii* again enters a tachyzoite state in which its complex organelles can rapidly secrete abundant proteins, e.g., microneme proteins, dense granules antigens and rhoptry protein. These proteins perform a variety of functions required to facilitate *T. gondii* parasitization, such as: immune escape, invasion, modification of parasitophorous vacuole, formation of intracellular vesicles, and assisting in entry into the host cell nucleus, in order for it to reproduce. As a result, *T. gondii* is often referred to as one of the “smartest” parasites as the species evolves. Among the many organelles of *T. gondii* and their secreted proteins, the rhoptry protein by the rhoptry play an important role in their parasitism and reproduction. ROP16 is a member of the Rhoptry protein family, which, together with ROP18, is currently considered one of the most important virulence factors for *T. gondii* invasion of the host. And it has been shown to modulate host cell signaling pathways, including the STAT family of transcription factors, which play important roles in cell proliferation, differentiation, and survival. In this study, we investigated the effects of ROP16 on A549 cells, a commonly used model of lung adenocarcinoma. Our results demonstrated that type I and III ROP16 proteins inhibited A549 cell proliferation, induced cell cycle arrest at the G1 phase, and induced apoptosis. These effects were accompanied by changes in the expression of cell cycle- and apoptosis-related proteins. In addition, type I and III ROP16 proteins reduced the invasion and migration of A549 cells, suggesting that ROP16 may also affect the metastatic potential of lung adenocarcinoma cells. Our findings are consistent with previous studies demonstrating that ROP16 can modulate the expression of genes involved in cell adhesion, migration, and invasion in other cell types. Moreover, we found that typeI and III ROP16 proteins could activate STAT3 for a prolonged period, whereas type II ROP16 protein had no such effect. STAT3 is a well-known transcription factor that plays important roles in many kind of cell survival, proliferation, and migration.^30–32^ Dysregulation of STAT3 signaling has been implicated in the development and progression of many cancers, including lung cancer.^33, 34^ Therefore, our findings suggest that ROP16 may be involved in the regulation of STAT3 signaling in lung adenocarcinoma cells. Furthermore, we investigated the effects of ROP16 on primary lung adenocarcinoma cells and found that type I and III ROP16 proteins still inducing cell cycle arrest and apoptosis. These findings suggest that ROP16 may be a potential therapeutic target for lung adenocarcinoma.

STAT3, which is closely correlated with cancer, can regulate downstream genes to induce proliferation and inhibit apoptosis of cancer cells. The mechanisms of this regulation may include epigenetic changes, such as phosphorylation, DNA methylation and chromosomal modification, as well as alterations in mitochondrial function. However, in certain conditions, STAT3 may play the role of tumor inhibitor, suppressing the progression of various cancers, including lung adenocarcinoma, glioblastoma, prostate cancer, colorectal cancer, thyroid cancer, breast cancer, and esophageal squamous cell carcinoma.^35^ ROP16 has serine/threonine kinase loci in its protein sequence and can activate STAT3 by phosphorylation. The phosphorylated STAT3 continues to activate downstream effectors and signal pathways for the proliferation and apoptosis of cancer cells. In our study, a similar phenomenon was observed in the lung adenocarcinoma.

The ROP16 gene of *T. gondii* is located on chromosome VIIb and encodes 707 amino acids, the full length of the gene is 3014 base pairs, while the full length of the cDNA is 2124 base pairs.^24^ By aligning the gene sequences, we found that the gene sequence of *T. gondii* varies among different strains, with the sequence of type I and III strains being more than 99% similar, the sequence similarity between type II strains and type I and III strains is 97%. Yamamoto et al. demonstrated using an in vitro detection system that the kinase activity of ROP16 is essential for the activation of STAT3, and that the polymorphism between I and II strains of *T. gondii* is the reason for the differences in the regulation of STAT3 activation by ROP16.^25^ Previous research has demonstrated that type I and III ROP16 can directly phosphorylate Tyr705 in STAT3 and Tyr641 in STAT6, in addition, the N-terminal portion of ROP16 interacts with STAT3.^36^ By comparing type I and II ROP16 Yamamoto et al. revealed that a single amino acid(L503S)substitution in the kinase domain determined the strain difference in terms of Stat3 activation.^25^ Recent studies have shown that ROP16, a secreted protein of *T. gondii*, has anti-tumor effects by improving host immunity, inhibiting tumor progression and vessel growth, inducing cell cycle arrest, and manipulating oncogene expressions. Previous studies have shown that ROP16 mediates partially Sh-Sy5y cells apoptosis and cell cycle arrest by directing Ser15/37 phosphorylation of P53.^19^ ROP16 has also been found to indicate that *T. gondii* suppresses plasmacytoid dendritic cell activation by mimicking IL-10‘s regulatory effects through an ROP16 kinase-dependent mechanism.^37^ In addition, ROP16I and III ameliorated inflammatory bowel diseases via inducing M2 phenotype of macrophages.^38^ Another study showed that ROP16 enhance host immune response by activating NK cells, CD8+ T cells, and macrophages.^39^ A study results establish that *T. gondii* preferentially invades tumor-associated antigen-presenting cells and restores their ability to trigger potent antitumor CD8(+) T-cell responses.^16^ These findings provide new insights into the anti-tumor mechanisms of *T. gondii* and its secreted proteins, and suggest that ROP16 could be a promising candidate for the development of new anti-cancer drugs.

In this study, we found that type I and III ROP16 can induce cell cycle arrest in G0/G1 phase in a p21-regulated manner in both lung adenocarcinoma cell lines and primary cells. P21 is a well-known inhibitor of the cell cycle and can arrest cell cycle progression in G0/G1 by inhibiting the activity of the cyclinD1-CDK6 complex.^40^ Inducing apoptosis in cancer cells is one of the most important anti-cancer therapies, in recent years, targeted therapies for cancer have been developed that focus on apoptosis-associated proteins, such as Bax and Bcl-2.^41^ At the same time, several studies have shown that the Bax/Bcl-2 cascade and the EGFR pathway have greater therapeutic potential and are very important in NSCLC.^42^ Our study demonstrated that ROP16 upregulated the pro-apoptosis protein Bax and downregulated the anti-apoptosis protein Bcl-2, leading to apoptosis of A549 cells. Additionally, ROP16 activated the p53-induced mitochondrial pathway of apoptosis, as evidenced by an increase in Caspase 9 and cleaved caspase 3. One of the key processes that contribute to cancer progression and metastasis is the epithelial-mesenchymal transition (EMT), during EMT, cancer cells can acquire mesenchymal characteristics, which are associated with increased motility, invasiveness.^43^ In this context, our study found that type I and III ROP16 can inhibit the invasive ability of these cells and suppress cancer metastasis, and act as a negative regulator of EMT and cancer progression. Our results suggest that ROP16 may provide new insights into the development of targeted therapies for cancer treatment.

Summarizing our discussion of ROP16 function, amino acid polymorphisms and STAT3 phosphorylation sites, in our next work we will first explore the effects of amino acid sequence differences among the three genotypes of ROP16 on lung adenocarcinoma cell phenotypes and STAT3 phosphorylation as a means of identifying the major amino acid sites responsible for the difference in function between type I/III ROP16 and type II ROP16 in lung adenocarcinoma cells. Secondly, we will construct full-length and truncated ROP16 expression vectors containing the major functional regions, express and purify ROP16 protein, and further investigate the inhibitory effect of ROP16 on lung adenocarcinoma cell growth at the exogenous level, and use this as a basis for attempting to design small-molecule peptide anti-tumor adjuvants based on the functional region of ROP16, and further validate its adjuvant anticancer effect in a mouse model of lung adenocarcinoma. In addition, in order to address the non-specificity of ROP16 protein, we propose that in the future, by further modifying the ROP16 protein and utilizing drug targeting delivery systems such as coupling magnetic nanoparticles, we can achieve precise delivery of ROP16 protein. Magnetic controlled nanorobots may be created by combining magnetic nanoparticles with ROP16, a substance that possesses anti-tumor action. This allows for very precise targeted therapy of lung adenocarcinoma with less negative impact on surrounding normal tissue. This may be utilized to confirm that the ROP16 inhibits lung adenocarcinoma cell proliferation in vivo.

In conclusion, our study demonstrates that type I/III ROP16 inhibits cell proliferation, induces cell cycle arrest and apoptosis, and reduces invasion and migration in lung adenocarcinoma cells. These effects are mediated, at least in part, by the activation of STAT3 signaling. Our findings suggest that ROP16 may be a potential therapeutic target for lung adenocarcinoma. Further studies are needed to fully understand the mechanisms by which ROP16 regulates these processes and to investigate the potential of ROP16 as a therapeutic target in vivo.

## Materials and methods

### Cell culture of A549 lung adenocarcinoma cells

The A549 human lung adenocarcinoma cell line was obtained from the Shanghai Cellular Research Institute, Chinese Academy of Science (Shanghai, China) and stored at the Key Laboratory of Western Featured Bioresources and Utilization, Ministry of Health, Ningxia University, China. The cells were identified by STR, mycoplasma tests were negative, and the cells were routinely cultured in Dulbecco’s Modified Eagle’s Medium (DMEM) supplemented with 10% fetal bovine serum (FBS) and passaged as needed.

### Selection of stable A549 cell lines with ROP16 overexpression

Gene sequences of three strains of *T. gondii*, namely, RH (type I), Me49 (type II), and VEG (type III), were obtained from the website https://toxodb.org/toxo/app, enzymatically cleaved and ligated with pHBLV-CMV-MCS-EF1-Zsgreen1-T2A-puro, His tags were added and lentiviral expression vectors were constructed, using pSPAX2, pMD2G and shuttle plasmid were used for lentiviral packaging. The lentiviral vector construction was provided by Hanbio Biotechnology Co., Ltd, and the PCR primers were produced by Shanghai Sangong Biotechnology Co., LTD. Lentiviral vectors overexpressing ROP16 were transfected with A549 cells using Lipofectamine3000 as a transfection reagent and screened with puromycin for 2 weeks to obtain stable cell lines overexpressing ROP16. Real-time PCR, Western blotting, and immunofluorescence assay were used to verify the successful transfection and expression of ROP16.

### A549 cell proliferation detection by CCK-8 assay

A549 cells, A549-HBLV cells,A549-RH ROP16 cells, A549-Me49 ROP16 cells, A549-VEG ROP16 cells in logarithmic phase were inoculated in 96-well plates at a density of 3 × 10^4^ cells/well. After incubation for 24 h, 48 h and 72 h respectively, CCK-8 was added and incubated for 2 h at 37°C, and the OD values were measured at a wavelength of 450 nm, CCK-8 reagent kit was purchased from Beijing Saiwen Creative Biotechnology Company.

### A549 cell cycle and apoptosis detection by Flow Cytometry

A549 cells, A549-HBLV cells and three strains A549-ROP16 cells in logarithmic phase were inoculated in 6-well plates at a density of 5.0 × 10^5^ cells/well and cells were collected after 48 h of incubation. Add pre-chilled 75% ethanol and fix overnight at 4°C. Centrifuge and discard the supernatant, wash the cells with an appropriate amount of PBS to remove the ethanol. Add DNA staining solution and permeabilization solution and incubate for 30 min at room temperature, protected from light, and then use a Flow Cytometer to detect the cell cycle. According to Annexin V-PE/7-AAD apoptosis kit, a blank tube was set to adjust the voltage of FSC, SSC, and fluorescence channel on the flow cytometer, and a single staining tube was set to adjust the compensation of fluorescence channel. 5 μL Annexin V-APC and 10 μL 7-AAD were added to the cells in each tube, the cells were incubated at room temperature in the dark for 5 min and the level of apoptosis analyzed by Flow Cytometry.

A549 cells, A549-HBLV cells and three strains A549-ROP16 cells in logarithmic growth stage were inoculated into 24-well plates with 5 × 10^4^ cells per well. After 48 h of culture, the cells were moistened with PBS, followed by 500 μL 4% paraformaldehyde in each well and fixed at room temperature for 30 min. Then, the cells were moistened with PBS, followed by 500 μL 0.3% TritonX-100(diluted with PBS) in each well. It was permeated at room temperature for 5 min, and then washed twice with PBS. TUNEL test solution was prepared according to the instructions for reagent use. 50 μL TUNEL test solution was added to the sample and incubated at 37°C for 60 min in the dark. After washing with PBS, the sample was sealed with DAPI and observed under fluorescence microscope. The TUNEL reagent kit was purchased from Shanghai Biyuntian biotechnology Company.

### A549 cell invasion detection by Transwell assay

The A549 cells, A549-HBLV cells and three strains A549-ROP16 cells in the logarithmic growth stage were inoculated into 24-well Transwell plates with 0.5 × 10^5^ cells per well, matrigel matrix glue was diluted with serum-free medium at 1:20 and added with 50 μL into the upper chamber of transwell plates and placed in CO_2_ incubator for 48 h. The medium was discarded and fixed with 4% paraformaldehyde 20 min. Dyeing with 0.5% crystal violet for 10 min and washing with PBS for 2–3 times, and the cell invasion was observed under microscope.

### Western blotting

The A549 cells, A549-HBLV cells and three strains A549-ROP16 cells were cultured to logarithmic growth phase, digested and collected by centrifugation, and each group of cells was added to protein lysate (RIPA: PMSF = 100 ː 1), followed by centrifugation at 12,000 × g for 15 min at 4◦C. The concentration of each group of proteins was measured by the BCA protein quantification kit.The denatured proteins were separated by 10% separationgel in SDS-PAGE and transferred to PVDF membrane followed by blocking in 5% of BSA on a shaker for 90 min. PVDF membranes were incubated with primary antibodies against β-actin, P21, CDK6 and CyclinD1 to detect the expression of cell cycle-related proteins in each group of cells, and incubated with primary antibodies against Bax, Bcl-2, p53, cleaved Caspase3, Caspase9 to detect the expression of apoptosis-related proteins in each group of cells, incubation was performed with anti-p-STAT3 as primary antibody, and STAT3 phosphorylation levels between the groups of cells have been detected. The antibody dilution ratio was 1:10 00 to 1:10 000 in all cases, and the incubation conditions were overnight at 4◦ C with 50 rpm shaking. The anti-β-actin was used as the control. The PVDF membrane was washed with TBST three times, followed by the horseradish peroxidaselabeled secondary antibody incubation with slight shaking for 90 min. The specific bands were visualized by enhanced chemiluminescence. All experimental data were analyzed by ImageJ software. The monoclonal antibodies used in this study were purchased from Abcam (USA), CST (USA), and Proteintech Company (USA).

### Real-time PCR

Total RNAs were extracted from the A549, A549-HBLV, and three strains A549-ROP16 cells by Trizol, then reversed into cDNAs according to the Takara Kit instructions (Takara, Japan). Quantitative detection of P21, CDK6 and CyclinD1,Bax, Bcl-2, Caspase9 of the A549, A549-HBLV and three strains A549-ROP16 cells was performed with SYBR-Green Premix Ex Taq kit (Takara, Japan). The primers used in this experiment were all synthesized by Shanghai Sangong Biotechnology Co., LTD., and the specific sequences are shown in Table 1. The measurements were completed with Roche Applied Science Light Cycler TM 480 instrument. The results were normalized by β-actin and the results were calculated using the 2^−△△CT^ method.

**Table 1.** PCR primer sequences.

Gene names	Sequences
*rop*16(F)	5´-TGGGCTCCTGAACTTGCGAAATC-3´
*rop*16(R)	5´-AGACGAACTCGAAGATTGCCAACC-3´
Bax(F)	5´-ATCAGAACCATCATGGGCTGGACA-3
Bax(R)	5´-AGCCCATCTTCTTCCAGATGGTGA-3´
Bcl-2(F)	5´-TTGTGGCCTTCTTTGAGTTCGGTG-3´
Bcl-2(R)	5´-ACTCACATCACCAAGTGCACCTAC-3´
Caspase9(F)	5´-TGGTGCTCAGACCAGAGATT-3´
Caspase9(R)	5´-ACGGGGTGGCATCTGGCTCG-3´
p21(F)	5´-GTCACTGTCTTGTACCCTTGTG-3´
p21(R)	5´-GGCGTTTGGAGTGGTAGAAA-3´
CDK6(F)	5´-TGCACAGTGTCACGAACAGACAGA-3´
CDK6(R)	5´-TTAGATCGCGATGCACTACTCGGT-3´
CyclinD1(F)	5´-CAAGCTCAAGTGGAACCTGG-3´
CyclinD1(R)	5´-GCGGATGATCTGTTTGTTCT-3´
β-actin(F)	5´-CACTGTGCCCATCTACGA-3´
β-actin(R)	5´-TGATGTCACGCACGATTT-3´

### Acquisition of primary human lung adenocarcinoma cells

With the informed consent of the patient and his family and the approval of the hospital ethics committee, post-surgical cancer tissues from lung adenocarcinoma patients were transferred to the laboratory at low temperature; the cancer tissues were picked and washed in 75% anhydrous ethanol for 30s; the tissues were washed 2–3 times in PBS. Tissue pieces cut to <1 mm^3^ were resuspended by adding 1 mL of 0.1% collagenase I solution and digested at 37°C for 60 mins in a shaker, and the digestion was terminated by adding an equal volume of F12 medium containing 20% FBS. After mixing, filter the cell suspension using a 200 mesh stainless steel sieve, centrifuge the filtrate at 1000rpm for 5 min, add 1 mL of red blood cell lysate to the sediment, centrifuge again and resuspend the cells with culture medium, inoculate into a 6-well plate at a suitable concentration for culture, scrape off the fibroblast area, add TrypLE™ Express enzyme (1×) to digest the epithelial cells, collect the digested suspension and centrifuge Continue culturing with F12 medium containing 20% FBS, 1/100 penicillin/streptomycin and 1/1000 amphotericin b until epithelial cells are present in the microscopic field.

### The regulation of ROP16 protein on primary human lung adenocarcinoma cells

Human lung adenocarcinoma primary cells were obtained from lung adenocarcinoma tissue samples and underwent over 3 passages. Lentiviral vectors overexpressing ROP16 were transfected with lung adenocarcinoma cells using Lipofectamine3000 as a transfection reagent, and the transfection efficiency was detected by real-time PCR and Western blotting. The levels of apoptosis-related protein, cell cycle-related protein and STAT3 phosphorylation in each group of cells were detected by real-time PCR and Western Blotting.

### Statistical analysis

Three independent experiments were performed to generate data for analysis. All results are presented as the mean value ± SEM. GraphPad Prism 9.0 software was used to analyze the data. Two-tailed unpaired t-tests or one-way ANOVA were used to determine the statistical significance between groups, and post-hoc Bonferroni correction was applied when necessary. In comparison to the control group, * denotes significant differences (*p* < .05), ** denotes highly significant differences (*p* < .01), and *** denotes extremely significant differences (*p* < .001). Compared to the empty vector control group, “a” denotes significant differences (*p* < .05), “b” denotes highly significant differences (*p* < .01), and “c” denotes extremely significant differences (*p* < .001).

## Supplementary Material

Supplemental Material

## Data Availability

The authors confirm that the data supporting the findings of this study are available within the article and its supplementary materials.

## Data availability statement

The datasets generated during and/or analyzed during the current study are available from the corresponding author on reasonable request.
