# Supplementary material for: The anticancer mechanisms of Toxoplasma gondii rhoptry protein 16 on lung adenocarcinoma cells
Source: Cancer Biol Ther. 2024 Aug 22;25(1):2392902. doi: 10.1080/15384047.2024.2392902 (PMC11346528; doi:10.1080/15384047.2024.2392902)
Supplement: Supplemental Material [file KCBT_A_2392902_SM5000.zip › Figure S1_Caption.docx]

Figure S1.Sequence alignment of Different Types of ROP16 Genes.The yellow area in the diagram represents the same sequence of three types, while the blue area represents the different sequence of three types.
