# Supplementary figures and images for: The anticancer mechanisms of Toxoplasma gondii rhoptry protein 16 on lung adenocarcinoma cells
Source: Cancer Biol Ther. 2024 Aug 22;25(1):2392902. doi: 10.1080/15384047.2024.2392902 (PMC11346528; doi:10.1080/15384047.2024.2392902)

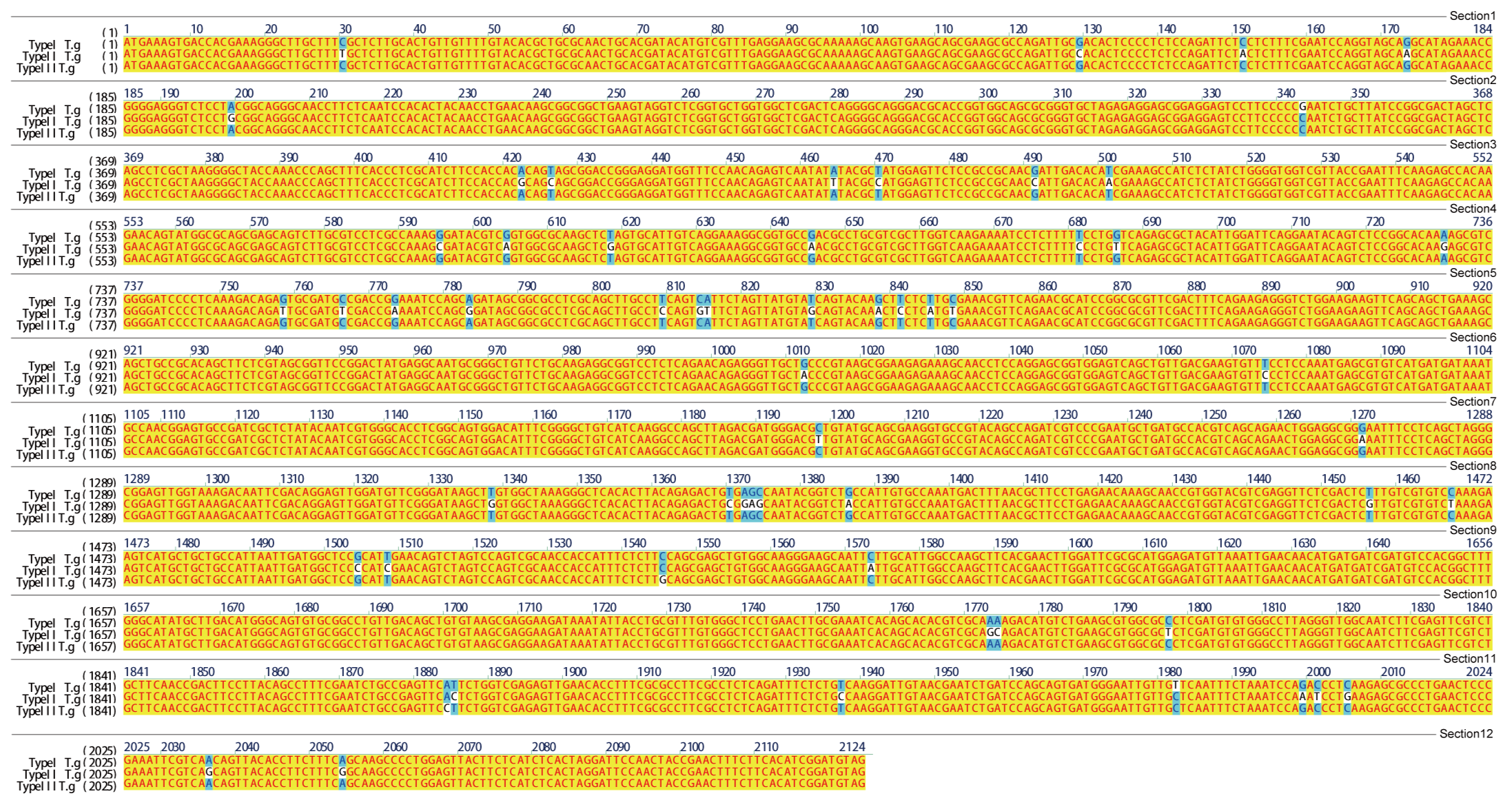

Supplement: Supplemental Material [file KCBT_A_2392902_SM5000.zip › Supplementary figure 1.tif]
